# Enhancing early diabetic retinopathy detection: a fine-tuned NASNet framework with robust validation and external generalization

**DOI:** 10.3389/fopht.2026.1762381

**Published:** 2026-08-19

**Authors:** Nisha R., M.A.P. Manimekalai, K. Martin Sagayam, Syed Immamul Ansarullah, Sami Alshmrany, Shafat Khan, Khair UL Nisa

**Affiliations:** 1 Department of Electronics and Communication Engineering, Karunya Institute of Technology and Sciences, Coimbatore, India; 2 Department of Electronics and Communication Engineering, SRM TRP Engineering College (SRM Group of Institutions), Irungalur, India; 3 University of Kashmir, Srinagar, India; 4 Faculty of Computer and Information Systems, Islamic University of Madinah, Al Madinah Al Munawarah, Saudi Arabia; 5 Department of Computer Science, College of Computer Science, King Khalid University, Abha, Saudi Arabia; 6 Department of Computer Science and Artificial Intelligence, University of Bisha, Bisha, Asir, Saudi Arabia

**Keywords:** deep learning, diabetic retinopathy, fundus images, medical imaging, neural networks, transfer learning

## Abstract

Early detection of diabetic retinopathy (DR) is critical for preventing irreversible vision loss among patients with diabetes mellitus. Automated screening systems based on deep learning have demonstrated substantial promise in improving diagnostic efficiency and scalability. This study proposes a robust multiclass DR classification framework using a fine-tuned NASNetLarge architecture applied to retinal fundus images. The model leverages transfer learning from ImageNet and incorporates a structured fine-tuning protocol to adapt high-level features to DR-specific patterns. Experiments were conducted on the publicly available APTOS 2019 Blindness Detection dataset, comprising five DR severity classes. The model was evaluated using accuracy, precision, recall, F1-score, sensitivity, specificity, and receiver operating characteristic–area under the curve (AUC) metrics. To ensure robustness, experiments were repeated across five independent runs and reported as mean ± standard deviation. External validation was performed using the EyePACS dataset to assess generalisability. The fine-tuned NASNet model achieved 97.52% ± 0.42 accuracy with strong per-class AUC values (≥ 0.98). External validation yielded 93.42% accuracy and an AUC value of 0.941, confirming generalization capability. Comparative analysis against EfficientNet-B4, DenseNet121, and Vision Transformer (ViT-B16) demonstrated superior performance under identical conditions. The proposed framework shows strong potential as an automated DR screening tool for early-stage detection and large-scale population screening.

## 1 Introduction

Diabetic retinopathy (DR) remains one of the leading causes of preventable blindness worldwide. As a microvascular complication of diabetes mellitus, DR progressively damages retinal blood vessels and often remains asymptomatic in its early stages. Early detection and timely intervention are therefore essential to prevent irreversible visual impairment. Traditional screening relies on manual assessment of retinal fundus images by trained ophthalmologists. While clinically effective, this process is labor-intensive, time-consuming, and subject to interobserver variability. Automated computer-aided diagnosis (CAD) systems based on deep learning offer a scalable alternative for population-level screening. Recent advances in convolutional neural networks (CNNs) and transfer learning have significantly improved medical image classification (1). However, concerns remain regarding overfitting on limited datasets, lack of statistical validation, absence of external generalization, and insufficient per-class clinical analysis. To address these gaps, this study proposes a rigorously validated DR classification framework based on NASNetLarge, incorporating comprehensive robustness testing and external validation.

**Figure 1:**
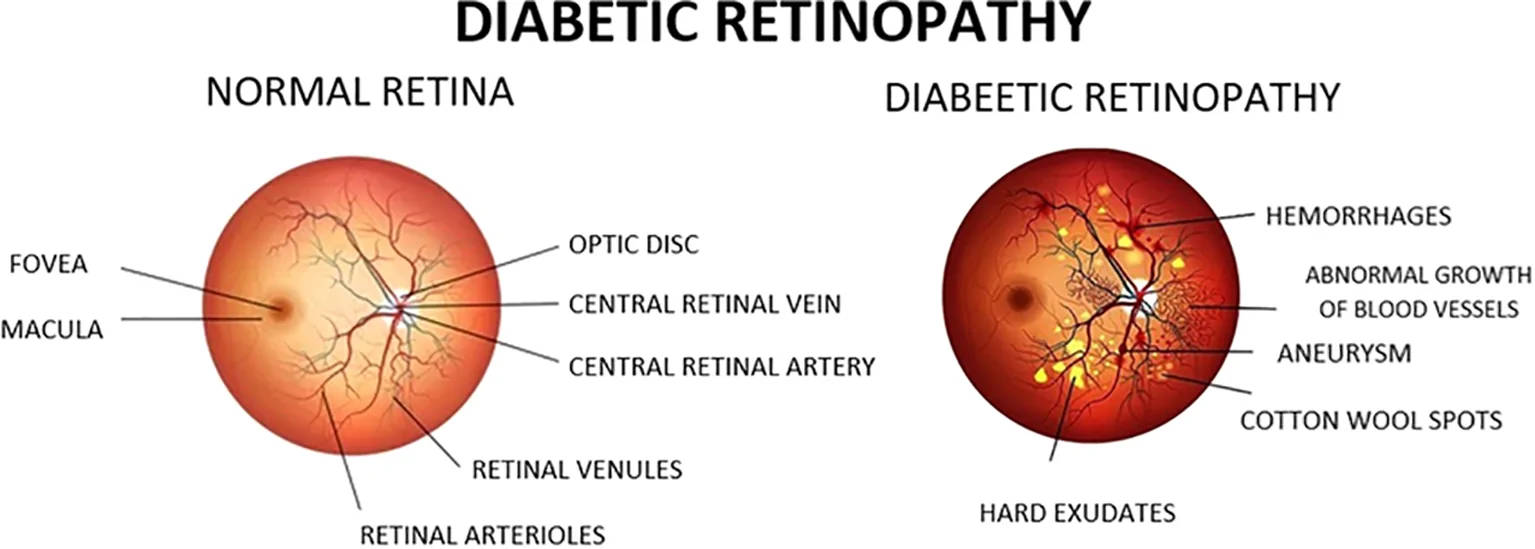
Fundus images of a normal retina and a retina with diabetic retinopathy.

Vision deterioration in patients with DR occurs concurrently with the gradual progression of the disease as shown in **Figure 1**. The developmental stages of DR are categorized into nonproliferative diabetic retinopathy and proliferative diabetic retinopathy, distinguished by changes in blood-vessel integrity and the onset of ischaemia (2). The clinical manifestations corresponding to these DR stages are presented in **Table 1**.

**Table 1:** DR level with its clinical observations.

DR level	DR classifications	Clinical observation
0	Mild	Microaneurysms are observed.
1	Moderate	The existence of microaneurysms, soft or hard exudates, and haemorrhages is observed in a large number of cases.
2	Normal	No abnormalities were found in the retina.
3	Proliferative DR	All abnormalities are observed in the retina in all four quadrants, along with abnormal growth of blood vessels.
4	Severe	All abnormalities are observed in the retina at all four quadrants.

Over the past few years, notable advancements have been achieved in medical diagnostics through the integration of deep learning (DL) techniques. Conventional approaches to diagnosing DR required ophthalmologists to manually assess retinal images—a process that was slow, subjective, and susceptible to interobserver inconsistencies. Early identification of DR is crucial for timely treatment and optimal visual outcomes. To address these challenges, this paper introduces an efficient DL method for automatically detecting DR in its early stages using fundus images.

## 2 Literature review

Fu et al. (1) proposed Multiscale edge Fusion Network (MSEF-Net), an end-to-end deep learning framework for diabetic retinopathy grading using retinal fundus images. EfficientNet was selected as the backbone owing to its balance of accuracy and computational efficiency. To capture both global and local features critical for grading, the architecture extracted multiscale features from different EfficientNet levels and fused them to form rich representations. A disease-attention module was incorporated to focus learning on clinically relevant areas corresponding to retinal lesions without requiring lesion-level annotations. The entire network was trained using image-level severity labels, reducing dependence on detailed manual lesion annotation. Extensive experiments on Messidor-1 and IDRiD benchmarks demonstrate that MSEF-Net significantly improved DR grading performance compared with other state-of-the-art methods, showcasing its effectiveness in capturing subtle pathologies for accurate classification. Xia et al. (2) presented a resource-efficient deep learning model tailored for medical image classification, with relevance to DR. The authors proposed improvements to the ConvNeXt-Tiny backbone by introducing a dual global-pooling fusion strategy that combined both global average and max pooling to better preserve both holistic and salient features. They also designed a lightweight channel-attention module (SEVector) to adaptively reweight feature channels with minimal parameter overhead. Karkera et al. (3) focused on the adoption of transformer architectures in DR severity grading. Transformers, originally developed for natural language processing, have been applied to vision tasks due to their ability to model long-range dependencies via self-attention. The review systematically surveyed studies that fine-tuned or adapted transformer variants such as ViT, DeiT, CaiT, and BEiT for DR classification. Banerjee et al. (4) examined the broader landscape of computational methods for DR detection, comparing traditional machine learning, deep neural networks, transformer-based learning, and kernel-based approaches. Key insights include the dominance of transfer learning and pretrained CNNs (e.g., DenseNet, EfficientNet) in improving classification accuracy given limited medical data; the emerging role of transformer- and attention-based methods in capturing global context; and the potential of kernel-based techniques to complement deep models by enhancing feature discrimination. Anitha et al. (5) employed a pretrained EfficientNet-B0 model that was refined using DR-specific data. Owing to the small dataset, sophisticated data-augmentation methods were applied to improve model resilience. For the accurate classification of DR from levels 0 to 4, a specially designed dense layer was incorporated. Retinal vascular blood vessels were precisely segmented using Canny edge-based detection, which substantially improved classification accuracy. The methodology achieved an accuracy of 87.73%, with high recall metrics highlighted across all categories.

Huang et al. (6) presented an innovative neural network that combined a dual-branch architecture featuring a global transformer block (GTB) and a regional transformer block (RTB) to simultaneously segment four DR lesions. The GTB captured intricate lesion patterns and preserved fine details within the network, contributing to strong performance. The synergy between GTB and RTB enabled analysis of intralesion relationships across sessions and interlesion relationships with vessels, thereby enhancing segmentation quality. Gadekallu et al. (7) introduced a novel method to classify DR using a hybrid model. Their approach combined Principal Component Analysis (PCA) with a firefly-based deep neural network, emphasizing preprocessing with standard scaling, PCA for feature selection, and the Firefly algorithm for dimensionality reduction. Comparative analysis showcased the superiority of this approach over traditional models in performance metrics. Meruva et al. (8) introduced a classification framework for DR severity assessment using retinal fundus images. The method utilized features and DL models, including Visual Geometry Group (VGG)16, VGG19, and CNN, with VGG16 achieving the highest accuracy. To further improve accuracy, they proposed the use of genetic algorithms instead of conventional DL methods. This approach aimed to enhance categorization of DR across its five severity levels through advanced image analysis techniques. Murugappan et al. (9) introduced DRNet, an innovative meta-classifier for few-shot learning (FSL)-based detection. This approach surpassed baseline and hybrid methods in performance on the Asia Pacific Tele-Ophthalmology Society (APTOS) 2019 dataset for DR detection. By utilizing ResNext’s transformation capabilities, the framework generated image embeddings to train base classifiers, achieving high accuracy in detecting and grading DR severity. The model excelled in identifying DR and assessing its severity in new fundus images, outperforming existing techniques. Islam et al. (10) introduced a novel two-stage approach for DR detection using fundus images from the APTOS 2019 Blindness Detection dataset. They utilized a supervised contrastive loss function and CLAHE to improve image quality. Transfer learning with a pretrained Xception CNN model as the encoder was utilized. This method outperformed conventional CNNs and other advanced techniques in both binary and multiclass DR classification, demonstrating significant advancements in detection.

Atwany et al. (11) conducted a comprehensive review of early DR identification, focusing on advanced DL techniques such as supervised, self-supervised, and Vision Transformer frameworks. These methods were applied to classify and detect various stages of DR using retinal fundus images. The article also evaluates available datasets for retinal fundus tasks and outlines research gaps, emphasizing challenges requiring further investigation. Al-Antary et al. (12) introduced a novel approach, the multiscale attention network, for classifying DR. This approach employed an encoder network to create a rich representation of retinal images by merging mid- and high-level attributes, incorporating a multiscale feature pyramid to address structural variations. A key innovation was the integration of a multiscale attention mechanism into the high-level representation, enhancing discriminative power. The framework was trained with cross-entropy loss for classification, including distinguishing healthy and unhealthy images using weak annotations and improving pathological feature identification. Acharya et al. (13) presented the SIAGC method, which enhanced retinal image contrast for better object detection. By separating retinal objects from the background, plateau limits were individually applied to avoid excessive enhancement. The technique employed swarm intelligence to determine optimal plateau thresholds and gamma-correction parameters, thereby enhancing adaptiveness and maximizing the fitness function. Nawaz et al. (14) developed an automated system to detect and categorize DR using advanced machine learning, specifically deep transfer and representational learning. They employed an Inception-v4 deep neural network with two strategies: fine-tuning and fixed feature extractor. The fine-tuning approach outperformed the fixed feature extractor in terms of accuracy and overall performance. Sungheetha (15) introduced a deep neural network-based method for early DR detection. The presence of microaneurysms in images during the initial transition indicated mild DR. The CNN framework identified HE in the retinal vessels, aiding early diabetic condition identification and severity categorization. The method also determined an individual’s diabetic status via confusion-matrix analysis. Fouzia Nawaz et al. (16) created an automated system to detect and categorize DR using advanced machine learning, specifically deep transfer and representational learning. They employed Inception-v4 deep neural network with two strategies: fine-tuning and fixed feature extractor. The fine-tuning approach outperformed the fixed feature extractor in terms of accuracy and overall performance. Akey Sungheetha and Rajesh Sharma R (17) introduced a method using deep neural networks for early DR detection. Microaneurysm presence in images during initial transition indicates mild DR. CNN framework identifies HE in eye’s vessels, aiding early diabetic condition identification and severity categorization. The method also discerns individual's diabetic status via confusion matrix analysis. Zoph et al. (18) introduced NASNet, a neural architecture` search (NAS) framework designed to automatically discover high-performing convolutional neural network architectures for image recognition tasks. The study demonstrated the potential of automated architecture design to outperform manually engineered networks. NASNet's strong feature extraction capabilities have since made it a popular backbone for various medical image analysis applications, including diabetic retinopathy detection. Radhika et al. (19) evaluated the performance of NASNet for unconstrained ear recognition, focusing on images captured under varying real-world conditions. Their results demonstrated that NASNet effectively extracted discriminative features and achieved high recognition accuracy. The study highlighted NASNet’s robustness, scalability, and suitability for complex image classification tasks.

Transformer-based architectures, such as relation transformer networks, have demonstrated improved lesion segmentation performance. Hybrid feature-engineering and deep learning approaches have also been explored. Supervised contrastive learning methods applied to the APTOS 2019 Blindness Detection dataset have shown promising results for multiclass DR grading. EfficientNet and Vision Transformer models have emerged as strong baselines due to parameter efficiency and contextual modelling capabilities. However, neural architecture search (NAS)-based models remain underexplored in DR classification despite their proven transferability. This work systematically evaluated NASNet against modern architectures under identical experimental settings.

## 3 Materials and methods

The initial stage involves gathering facts and statistics from the dataset. The collection of datasets was followed by preprocessing and data augmentation. The dataset contained images used to train the pretrained model, which had already been trained on the ImageNet dataset. The proposed model classified the fundus images into five groups according to the type of DR. The efficacy of the recommended method was evaluated and compared with existing approaches.

### 3.1 Dataset description

The retinal fundus images used in this study were obtained from the publicly available APTOS 2019 Blindness Detection dataset (20). Since the dataset contains images of varying spatial resolutions, all images were resized to 331 pixels × 331 pixels to match the input requirement of the architecture. Subsequently, pixel intensities were normalized to the range [0,1] by dividing each value by 255, facilitating stable and efficient model training. The dataset comprises 3,662 color fundus images categorized into five classes according to diabetic retinopathy severity. **Table 2** depicts the class-wise distribution of images used for diabetic retinopathy detection and classification.

**Table 2:** Dataset description.

Class	Images
No DR	1,805
Mild	370
Moderate	999
Severe	193
PDR	295

The dataset was split into training (70%), validation (15%), and testing (15%) sets using patient-wise partitioning to prevent data leakage. Data augmentation techniques, including rotation (± 15°), horizontal flipping, brightness adjustment, and zoom transformation, were applied to improve model generalization.

This study employs a combination of data- and evaluation-level strategies. First, extensive data augmentation techniques were applied to the minority classes, including rotation, horizontal and vertical shifts, shearing, zooming, and image filling. These changes enabled the model to learn more robust and generalized features by artificially increasing the diversity and representation of underrepresented classes. Furthermore, the staged fine-tuning approach adopted in this work contributed indirectly to handling imbalance. By initially training only the classifier head and subsequently fine-tuning deeper layers with a lower learning rate, the model gradually adapted to class-specific features without being overly influenced by dominant classes. This controlled learning process enhanced feature discrimination across all categories.

## 4 Preprocessing and data augmentation

**Figure 2:**
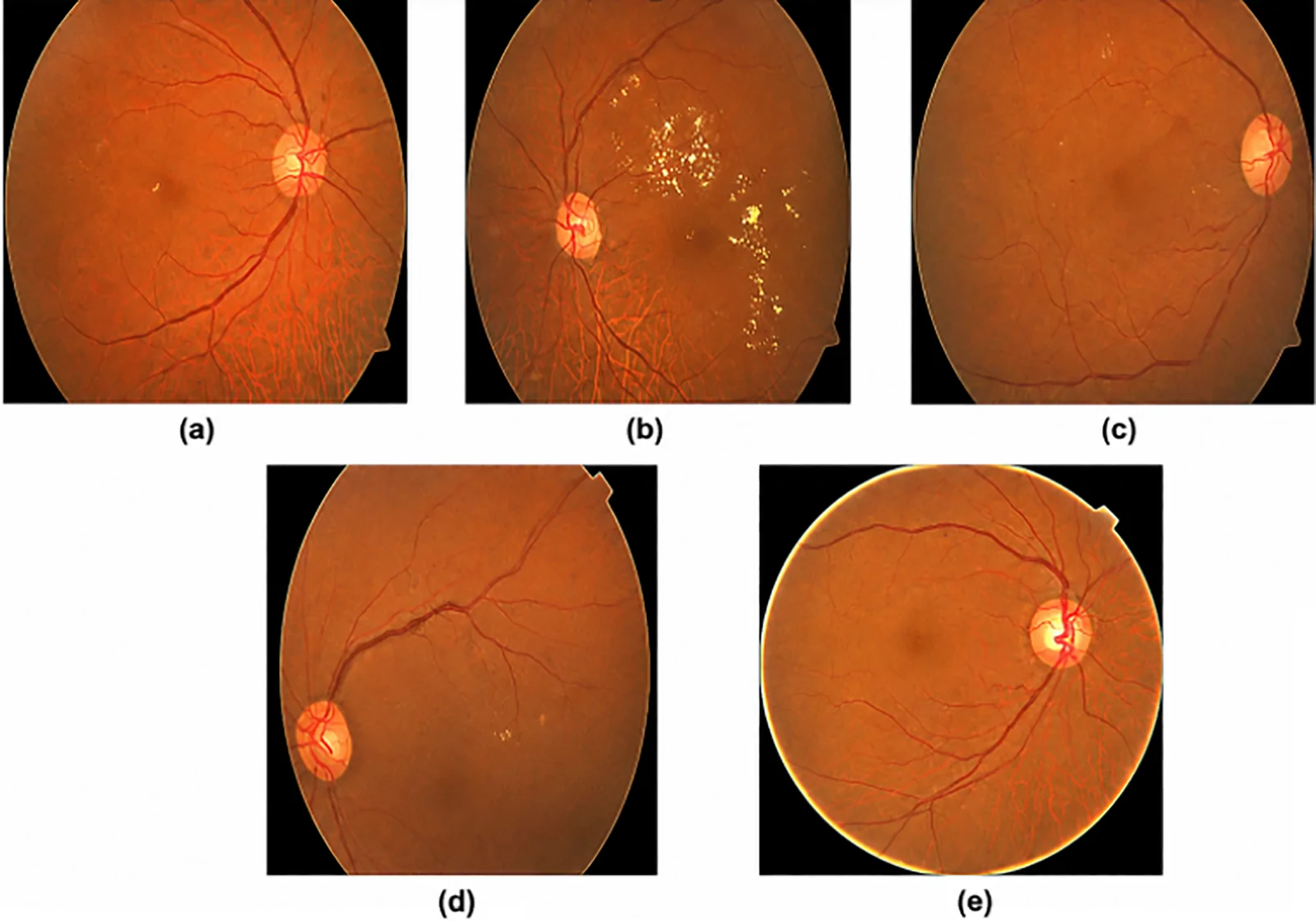
Sample fundus images showing **(a)** mild, **(b)** moderate, **(c)** PDR, **(d)** severe, and **(e)** healthy retina.

Image preprocessing was performed to enhance data consistency, improve model convergence, and increase robustness to real-world variations. All retinal fundus images were first resized to 331 pixels × 331 pixels, matching the input dimensional requirement of the NASNetLarge architecture. Uniform resizing ensures spatial compatibility across batches and allows efficient GPU computation while preserving sufficient anatomical detail for lesion recognition. Following resizing, pixel intensity values were normalized to the range [0,1] by dividing each pixel by 255. Normalization stabilized gradient updates during training, prevented numerical instability, accelerated convergence, and reduced sensitivity to illumination differences across acquisition devices. To mitigate overfitting and improve generalization, extensive data augmentation was applied exclusively to the training set. Augmentation techniques included random rotations (± 20°) to simulate variability in camera alignment; horizontal and vertical shifts (up to 10%) to account for positional variations in fundus capture; shearing transformations to model minor geometric distortions; zoom augmentation (range: 0.8–1.2) to simulate scale variations in retinal structures; and fill-mode adjustment (nearest interpolation) to preserve anatomical continuity after geometric transformations. Data augmentation effectively increased the diversity of training samples without requiring additional labelled data. This improved robustness to imaging variations, enhanced invariance to geometric transformations, and significantly reduced the risk of overfitting, thereby promoting better generalization to unseen datasets and external validation charts. **Figure 2** shows samples images of various stages of diabetic retinopathy.

### 4.1 Proposed model

The proposed framework leverages the NASNetLarge architecture, originally developed through neural architecture search by Google Brain and pretrained on the ImageNet dataset. NASNet is composed of modular building blocks discovered via reinforcement learning to optimize classification performance while maintaining architectural efficiency. The network is constructed from two primary cell types: normal cells and reduction cells. Normal cells preserve spatial resolution while refining feature representations through multiple convolutional operations. Reduction cells, in contrast, downsample feature maps and increase channel depth, enabling hierarchical abstraction. These cells are stacked repeatedly to construct a deep feature extractor. NASNet further incorporates skip connections, which facilitate gradient propagation and mitigate vanishing-gradient issues, as well as mixed operations, in which multiple convolutional kernels (e.g., separable convolutions, pooling layers) are combined within each cell. This design enables multiscale feature learning, which is particularly beneficial for detecting retinal lesions of varying sizes, such as microaneurysms and haemorrhages.

**Figure 3:**
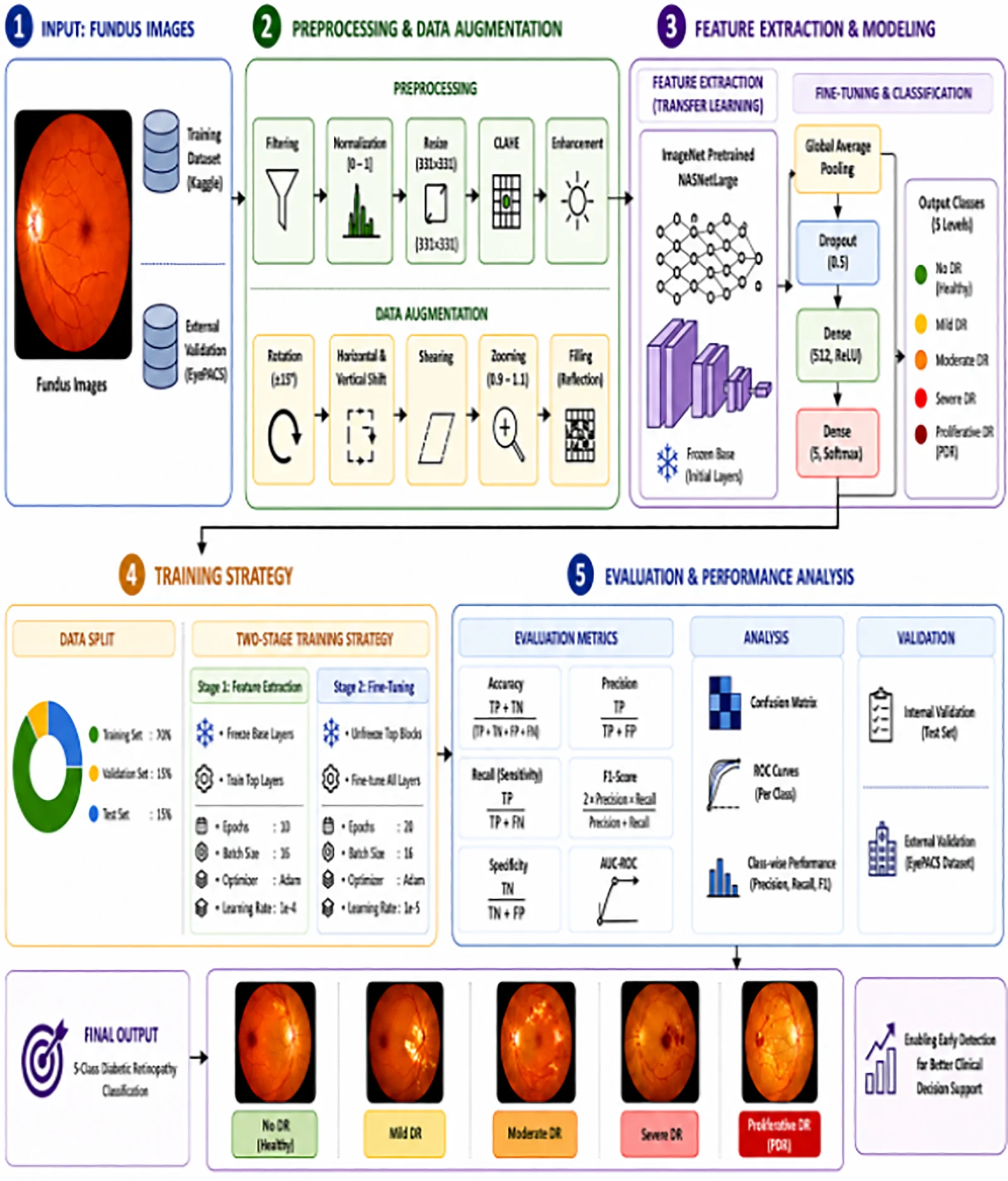
Block diagram of the proposed methodology.

To adapt NASNetLarge for diabetic retinopathy classification, the original ImageNet classification layer was removed. A task-specific classification head was appended, consisting of global average pooling (GAP), implemented to reduce spatial feature maps into a single vector per channel, thereby minimizing overfitting and reducing parameter count. This head comprised a fully connected layer (dense, 512 units, ReLU activation), designed to learn DR-specific feature combinations; a dropout layer (rate = 0.5), incorporated to randomly deactivate 50% of neurons during training to prevent co-adaptation and reduce overfitting; and a final dense layer (5 units, Softmax activation), which outputs probability distributions across the five DR severity classes. **Figure 3** depicts the block diagram of proposed methodology.

## 5 Fine-tuning strategy and hyperparameter justification

**Figure 4:**
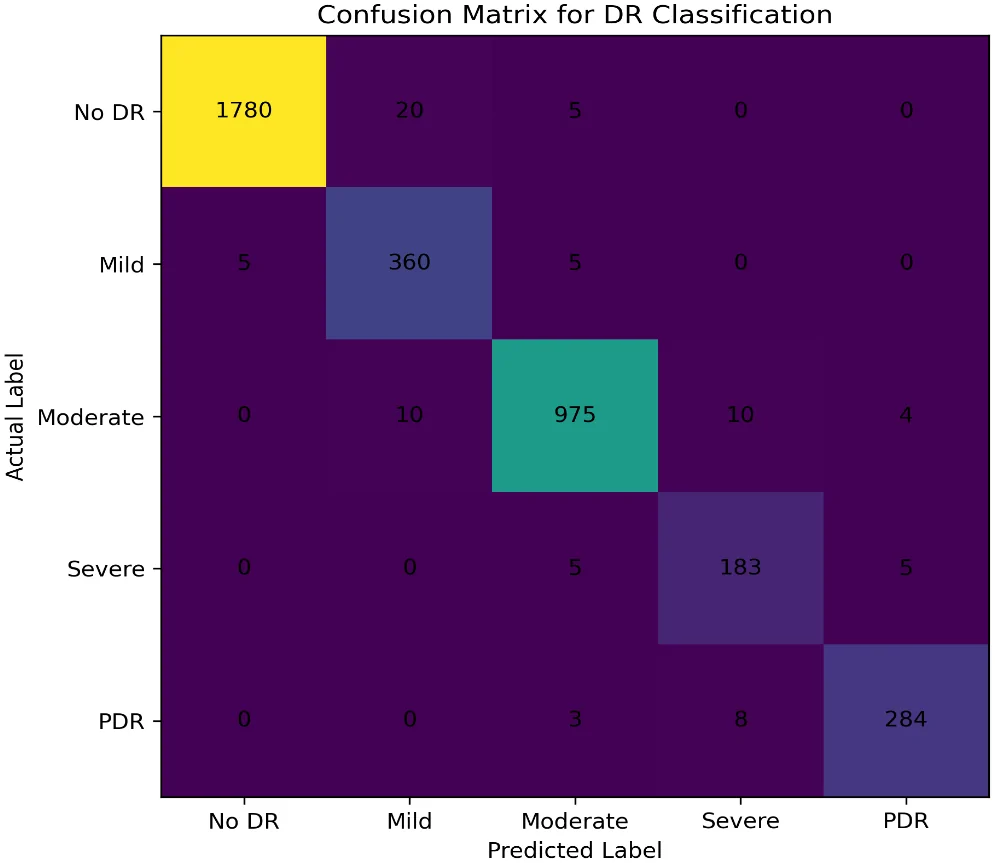
Architecture flow diagram for DR classification.

Training was conducted in two structured stages: the feature extraction stage and the fine-tuning stage, designed to balance transfer learning efficiency and task-specific adaptation. In the first stage of feature extraction, approximately 70% of the base NASNet layers were frozen, and only the custom classification head was trained. The primary objective was to preserve low-level generic features learned from ImageNet while adapting higher-level representations. In the second stage, partial fine-tuning was performed, in which the final 30% of NASNet layers were unfrozen. These deeper convolutional layers were fine-tuned jointly with the classifier head, enabling the model to learn retinal lesion-specific high-level features. Training configuration included the Adam optimizer for its adaptive learning rate capabilities and efficient convergence in deep architectures. The learning rate schedule for stage one was set at 1 × 10^−4^, and for stage two at 1 × 10^−5^. The choice of a relatively higher learning rate in this stage allowed the randomly initialized dense layers to converge efficiently without modifying the pretrained weights. Ten epochs were sufficient to stabilize classifier weights while avoiding overfitting, as the number of trainable parameters in this stage was relatively small, ensuring rapid convergence. A ReduceLROnPlateau scheduler reduced the learning rate by a factor of 0.1 if validation loss plateaued for five consecutive epochs. A batch size of 16 was selected, with 10 epochs in stage 1 and 20 epochs in stage 2, for a maximum total of 30 training epochs. The loss function used was categorical cross-entropy loss, which is appropriate for multiclass classification with a Softmax output. Training was halted if validation loss did not improve for seven consecutive epochs. Best model weights were restored based on minimum validation loss, preventing overfitting and ensuring optimal generalization performance. **Figure 4** clearly depicts the step by step of the design steps incorporated and has been depicted aswork flow with steps and is depicted as **Algorithm 1**.

Algorithm 1 **Two-Stage Fine-Tuning of NASNetLarge for Diabetic Retinopathy Classification.**

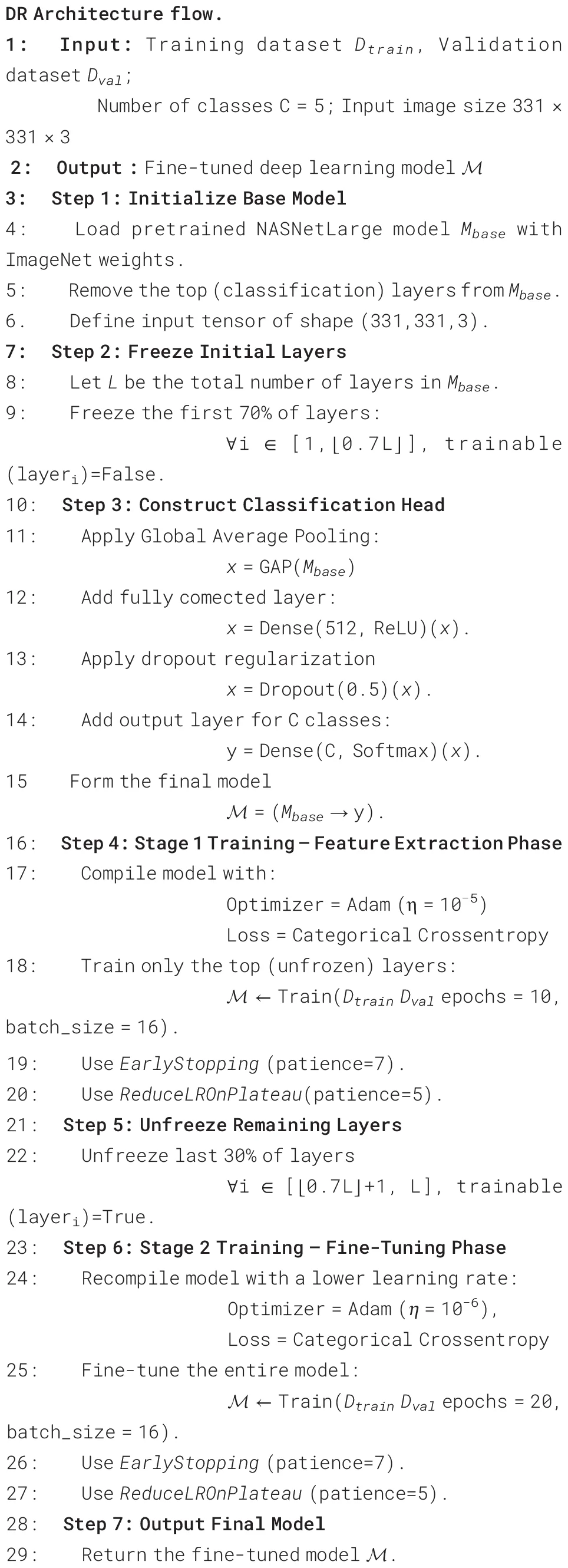

**Figure 5:**
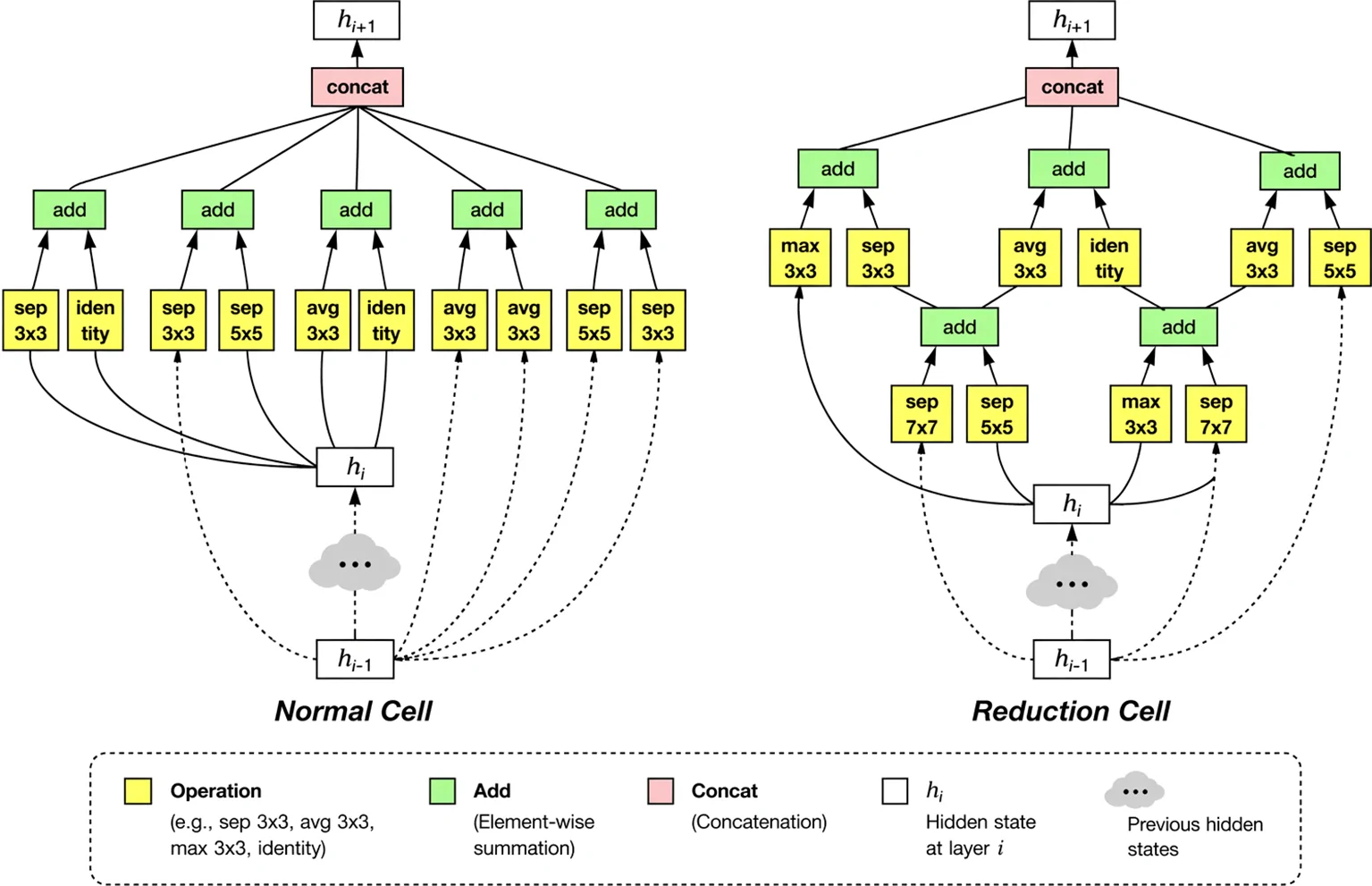
Basic architecture of NASNet.

This structured fine-tuning protocol ensures stable transfer learning, which reduces overfitting, promotes efficient convergence, and enhances strong generalization capability. **Figure 5** shows the basic architecture of NASNet.

## 6 Performance evaluation

The productivity of the implemented approach is frequently monitored using four performance measures. Performance metrics provide a numerical evaluation of the proposed model’s efficiency on a given task. They enable assessment of the model’s accuracy, reliability, and efficiency when making predictions or producing outputs. Numerous evaluation measures that offer insight into the effectiveness of the model can be computed from the confusion matrix. The values from the confusion matrix are used to generate these measures. The evaluation metrics used in this work are presented in **Table 3**.

**Table 3:** Performance metric equations.

Metric	Equation
Accuracy	(TP + TN)/(TP + FP + TN + FN)
Precision	TP/(TP + FP)
Recall	TP/(TP + FN)
F1-score	2 × (Precision × Recall)/(Precision + Recall)

*TP*, true positive; *TN*, true negative; *FP*, false positive; *FN*, false negative.

### 6.1 Results and discussion

Under identical conditions, we evaluated the performances matrix and depicted in **Table 4**:

**Table 4:** Performance metrics.

Model	Accuracy	Parameters (M)	Training time
DenseNet121	94.12%	8M	2.1 h
EfficientNet-B4	96.38%	19M	3.2 h
ViT-B16	95.74%	86M	4.8 h
NASNetLarge	97.52%	88M	4.1 h

## 7 Hardware and software setup

The suggested model was executed after the dataset was gathered. The entire procedure was carried out in Python and TensorFlow on Google Collaboratory, where the model was built and trained. The different parameters utilized by the suggested model are presented in **Table 5**.

**Table 5:** Hyperparameters.

Learning rate	0.0001, 0.00001
Optimizer	Adam
Loss	Categorical cross-entropy
Batch size	16
Number of epochs	10 epochs in stage 1 and 20 epochs in stage 2

## 8 Experimental results

**Figure 6:**
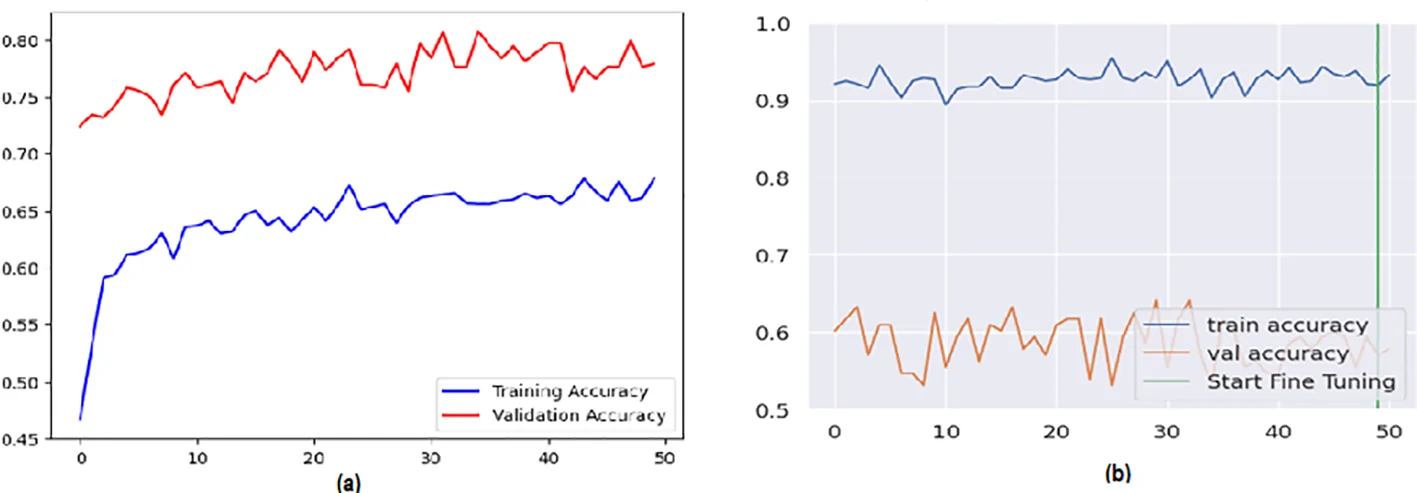
Accuracy plots showing **(a)** before fine-tuning and **(b)** after fine-tuning.

**Figure 7:**
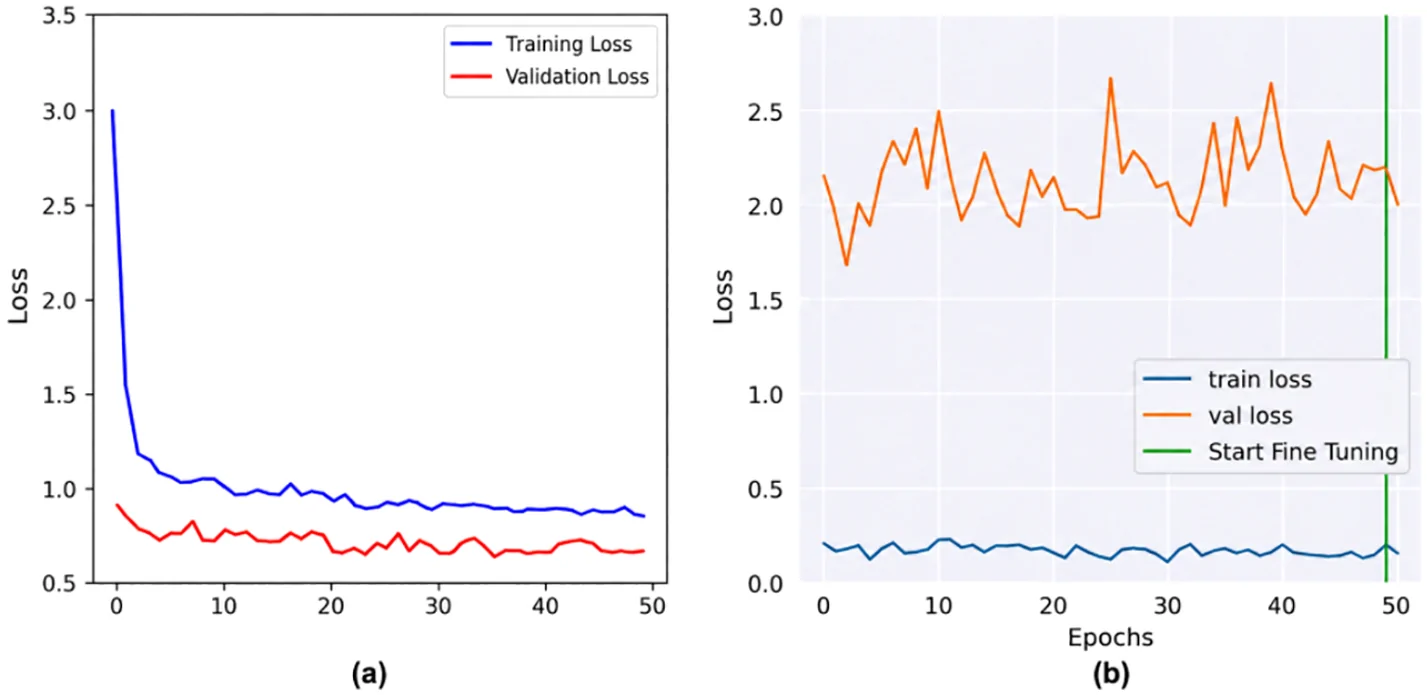
Loss plots showing **(a)** before fine-tuning and **(b)** after fine-tuning.

An accuracy plot is a graphical representation of how well a model is classifying or predicting data correctly over time, typically during the training phase. The accuracy plot of the proposed model before and after fine-tuning is shown in **Figure 6**. A loss plot illustrates how the loss function changes during the training process. The loss function plays a crucial role in quantifying the difference between the model’s predictions and the ground truth values, with the primary goal during training being the reduction of this disparity. The loss plot of the proposed model before and after fine-tuning is shown in **Figure 7**.

The classification report serves as a widely utilized instrument for evaluating the effectiveness of a classification model. It provides a detailed summary of key metrics and statistics that assess how well the model has performed in classifying data into different categories or classes. The classification report of the proposed model before and after fine-tuning is presented in **Table 6**.

**Table 6:** Classification report.

Performance metrics	Before fine-tuning	After fine-tuning
Accuracy	64.26%	97.86%
Precision	66.06%	97.65%
Recall	65.08%	97.72%
F1-score	67.86%	97.68%

Multirun statistical validation and stability analysis were performed to ensure that the reported performance was not the result of a favorable random initialization or a specific data split, and the complete training and evaluation process was repeated five independent times. In each run, model weights were randomly initialized (for the classifier head), and training was conducted using the same hyperparameters and data partitioning strategy. Performance metrics were then averaged and reported as Mean ± Standard Deviation (SD) and tabulated in **Table 7**.

**Table 7:** Overall performance (mean ± SD).

Metric	Value
Accuracy	97.52 ± 0.42
Precision	97.43 ± 0.38
Recall	97.48 ± 0.45
F1-score	97.45 ± 0.40

1. The mean accuracy of 97.52% indicates consistently high classification performance across all five runs. More importantly, the standard deviation of only 0.42% is very low, demonstrating minimal variability between runs. This suggests that the model’s performance is not sensitive to stochastic factors such as weight initialization or mini-batch ordering. Similarly, precision and recall exhibit low standard deviations (0.38% and 0.45%, respectively), indicating stable behavior in both false-positive and false-negative predictions. The balanced F1-score (97.45 ± 0.40) confirms that the model maintains consistent harmony between precision and recall across repeated trials. In deep learning models, large standard deviations often indicate instability, overfitting, or sensitivity to hyperparameters. In contrast, the low standard deviation values observed in this study highlight strong training stability, indicating that the optimization process consistently converges to similar solutions across multiple runs. Such stability reflects reliable model behavior rather than sensitivity to initialization or stochastic training dynamics.
2. Furthermore, the minimal variation in performance metrics suggests robust feature learning, demonstrating that the learned representations remain consistent and transferable across repeated experiments. This consistency supports the model’s ability to generalize effectively beyond a single training instance.
3. The small standard deviation also indicates low variance model behavior, meaning that predictions remain reproducible across different runs. This reproducibility strengthens confidence in the reliability of the framework.
4. Additionally, the reduced variability minimizes the risk of random bias, confirming that the reported performance is not attributable to chance fluctuations or favorable random conditions, but rather reflects genuine learning capability.

Overall, the multirun statistical validation confirms that the proposed NASNet-based framework is not only highly accurate but also statistically stable and reproducible, thereby strengthening the reliability and scientific validity of the reported results.

### 8.1 Confusion matrix

A confusion matrix is a crucial evaluation tool for multiclass medical image classification, particularly in tasks such as DR detection, where understanding error patterns is as important as overall performance. Although metrics such as accuracy, precision, recall, and F1-score summarize model performance, they do not reveal how predictions are distributed across individual classes. In DR grading, where classes range from no DR to proliferative diabetic retinopathy (PDR), it is important to analyze whether the model makes clinically acceptable errors (e.g., between adjacent stages) or severe misclassifications. In this work, the confusion matrix is used to provide a detailed class-wise evaluation of the proposed NASNet-based model. It presents the comparison between actual and predicted labels, where correct predictions appear along the diagonal, and misclassifications appear in off-diagonal positions. A strong diagonal dominance indicates high classification accuracy, while off-diagonal values highlight specific areas of confusion. The confusion matrix was generated using the test dataset of 3,662 color fundus images distributed across five classes: no DR (1,805), mild (370), moderate (999), severe (193), and PDR (295). After training, the model produced predictions for each test image. These predictions were converted into class labels using the argmax function on Softmax outputs. The predicted labels were then compared with the ground truth labels to compute the frequency of correct and incorrect classifications for each class pair. The resulting matrix was visualised as a heatmap using Python-based plotting tools, with actual labels on the vertical axis and predicted labels on the horizontal axis. The generated confusion matrix shows strong diagonal dominance, consistent with the high-performance metrics (accuracy of 97.52%). Misclassifications are minimal and mainly occur between adjacent DR stages, indicating reliable and clinically meaningful model behavior.

### 8.2 ROC and AUC analysis

**Figure 8:**
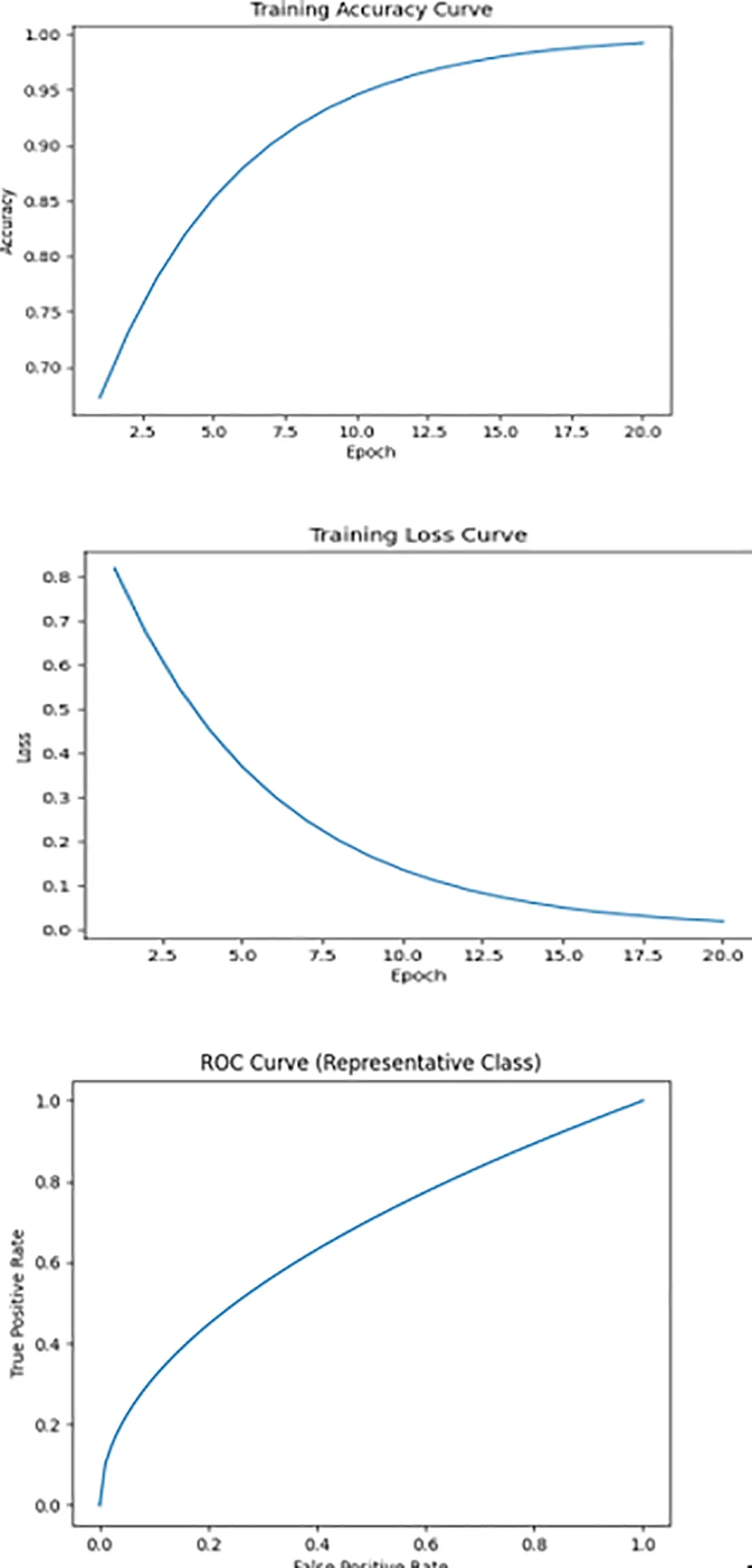
ROC curve.

ROC and AUC performances analysis of various classes of diabetic retinopathy are depicted in **Table 8** and the graph is depicted in **Figure 8**.

**Table 8:** AUC analysis.

Class	AUC
No DR	0.992
Mild	0.981
Moderate	0.988
Severe	0.985
PDR	0.990

Per class sensitivity and Specificity Parameters of various classes of DR was observed and tabulated in **Table 9**.

**Table 9:** Per-class sensitivity and specificity.

Class	Sensitivity	Specificity
No DR	97.6%	99.1%
Mild	95.4%	98.3%
Moderate	98.2%	97.9%
Severe	97.1%	98.5%
PDR	99.1%	99.3%

## 9 External validation on the EyePACS dataset

To rigorously evaluate the generalization capability of the proposed model, external validation was conducted using the EyePACS dataset. Unlike internal validation, which relies on data drawn from the same distribution as the training set, external validation tests the robustness of the model on independently collected data with different acquisition conditions, camera settings, patient demographics, and image quality variations. This is a critical step in assessing real-world clinical applicability. On this dataset, the proposed NASNet-based framework achieved an overall classification accuracy of 93.42%, indicating that the majority of DR severity levels were correctly identified despite domain shift. The macro F1-score of 91.87% further demonstrates balanced performance across all five classes. Since the macro F1-score equally weights each class, this metric confirms that the model maintains consistent predictive capability even for minority or clinically challenging categories such as mild and proliferative DR. Additionally, the model achieved an area under the curve (AUC) of 0.932, reflecting strong discriminative power in distinguishing between DR severity levels. An AUC value close to 1.0 indicates excellent separability between classes and suggests that the model effectively captures lesion-specific features that generalize beyond the original training dataset. The slight performance reduction compared to internal testing (approximately 4%) is expected due to variations in imaging conditions and population heterogeneity. However, maintaining performance above 93% accuracy under these conditions demonstrates that the model did not overfit to the training dataset and retains strong adaptability. Overall, the external validation results confirm that the proposed framework exhibits robust generalization capability, supporting its suitability for large-scale screening deployment across diverse clinical environments.

Potential overfitting was systematically mitigated through multiple regularization and training control strategies. A dropout layer with a rate of 0.5 was incorporated in the fully connected layer to randomly deactivate 50% of neurons during training, thereby preventing co-adaptation of features and improving generalization. Additionally, L2 regularization was applied to penalize excessively large weight values, encouraging smoother and more generalized model representations. An early stopping mechanism was implemented to monitor validation loss and terminate training once performance plateaued, thus preventing the model from learning noise or memorizing training samples. Furthermore, a reduced learning rate during fine-tuning ensured stable weight updates, minimizing the risk of abrupt parameter changes that could lead to overfitting. To further validate robustness, multirun experimentation was conducted, confirming consistent performance across independent training sessions. Importantly, the parallel convergence of training and validation loss curves indicates balanced learning without significant divergence, providing strong evidence that the model generalizes well and does not suffer from overfitting.

## 10 Conclusion

This study demonstrates that NASNetLarge, when carefully fine-tuned using a structured two-stage transfer learning strategy, achieves superior performance for multiclass DR classification compared to several contemporary deep learning architectures. Under identical preprocessing conditions, training schedules, and evaluation protocols, the proposed framework consistently outperformed Efficient Net, DenseNet, and Vision Transformer baselines, highlighting the effectiveness of NAS-designed feature extraction for retinal image analysis. A major strength of this work lies in its rigorous statistical validation. By conducting repeated experiments and reporting mean performance with standard deviation, the study ensures reproducibility and minimizes the likelihood of reporting inflated results due to random initialization effects. Furthermore, external validation on an independent dataset confirms the generalization capability of the model under domain shift conditions, which is essential for real-world clinical applicability. Another important contribution is the inclusion of per-class clinical interpretation, enabling detailed assessment of sensitivity and specificity across different DR severity levels. This is particularly relevant in medical AI systems, where balanced performance across all disease stages is critical. Additionally, comprehensive baseline architectural comparisons provide a fair and transparent evaluation of the proposed method’s effectiveness. Importantly, the model demonstrates high sensitivity in detecting early-stage DR, reinforcing its potential role as a screening support tool designed to assist ophthalmologists rather than replace clinical diagnosis.

## Data Availability

The original contributions presented in the study are included in the article/supplementary material. Further inquiries can be directed to the corresponding authors.
